# Tuning the Electronic Structure of a Novel 3D Architectured Co-N-C Aerogel to Enhance Oxygen Evolution Reaction Activity

**DOI:** 10.3390/gels9040313

**Published:** 2023-04-07

**Authors:** Chunsheng Ni, Shuntian Huang, Tete Daniel Koudama, Xiaodong Wu, Sheng Cui, Xiaodong Shen, Xiangbao Chen

**Affiliations:** 1 College of Materials Science and Engineering, Nanjing Tech University, Nanjing 210009, China; 2 AECC Beijing Institute of Aeronautical Materials, Beijing 100095, China

**Keywords:** aerogel, Co-N-C, DFT calculations, electrocatalysis, oxygen evolution reaction

## Abstract

Hydrogen generation through water electrolysis is an efficient technique for hydrogen production, but the expensive price and scarcity of noble metal electrocatalysts hinder its large-scale application. Herein, cobalt-anchored nitrogen-doped graphene aerogel electrocatalysts (Co-N-C) for oxygen evolution reaction (OER) are prepared by simple chemical reduction and vacuum freeze-drying. The Co (0.5 wt%)-N (1 wt%)-C aerogel electrocatalyst has an optimal overpotential (0.383 V at 10 mA/cm^2^), which is significantly superior to that of a series of M-N-C aerogel electrocatalysts prepared by a similar route (M = Mn, Fe, Ni, Pt, Au, etc.) and other Co-N-C electrocatalysts that have been reported. In addition, the Co-N-C aerogel electrocatalyst has a small Tafel slope (95 mV/dec), a large electrochemical surface area (9.52 cm^2^), and excellent stability. Notably, the overpotential of Co-N-C aerogel electrocatalyst at a current density of 20 mA/cm^2^ is even superior to that of the commercial RuO_2_. In addition, density functional theory (DFT) confirms that the metal activity trend is Co-N-C > Fe-N-C > Ni-N-C, which is consistent with the OER activity results. The resulting Co-N-C aerogels can be considered one of the most promising electrocatalysts for energy storage and energy saving due to their simple preparation route, abundant raw materials, and superior electrocatalytic performance.

## 1. Introduction

Hydrogen energy has been considered one of the most promising renewable energy sources currently due to its ease of use and lack of pollution. Hydrogen generation through water electrolysis, based on the future objective of reaching carbon neutrality or perhaps carbon negative, has considerable potential for development and implementation among the different methods of hydrogen preparation [1,2]. The water electrolysis reaction consists of two half-reactions, one is the hydrogen evolution reaction (HER) in the cathode region, and the other is the oxygen evolution reaction (OER) in the anode region. Different from the two-electron transfer reaction of HER, OER is a four-electron proton coupling reaction, which requires higher energy (higher overpotential) to overcome the kinetic barriers of OER [3]. In recent decades, various catalysts have been developed to improve the reaction kinetics and stability of electrodes. Noble metal-based oxides are widely used OER electrocatalysts, such as RhIr alloys [4], IrO_2_ [5], and RuO_2_ [6]. The high stability and catalytic activity of noble metal catalysts provide low overpotential and long-term stability in highly corrosive acidic or alkaline electrolytes. However, the expensive prices and scarce stocks of noble metal catalysts limit their application. Currently, many novel nanomaterials, such as transition metals [7], non-metallic carbon-based materials [8], and nano-carbon hybrids [9], have been investigated as alternatives to precious metal catalysts. Qiu et al. [10] used chemical vapor deposition to fabricate a three-dimensional graphene-coated nanoporous metallic nickel catalyst with an OER overpotential as low as 0.36 V at a current density of 10 mA/cm^2^. Lyu et al. [11] used pyrolysis to prepare a Co-N-C catalyst with Co nanoparticles encased in an N-doped carbon layer that had a low OER overpotential of 0.41 V at a current density of 10 mA/cm^2^. In addition, increasing the specific surface area of the catalyst can expose more catalytic active sites, which is another effective method to enhance the OER activity. The specific surface area of catalysts can be considerably increased by altering the morphology or loading on porous substrates [12].

Aerogel is a nanomaterial made up of nanoparticles, nanowires, or nanosheets that has a three-dimensional porous network-like structure. It is widely employed in the domains of thermal insulation, adsorption, catalysis, and energy storage due to its coherent pore structure and high specific surface area [13,14,15]. Aerogels used as electrocatalysts for OER reactions can be broadly classified into two categories: catalyst barriers and aerogel-structured catalysts. Aerogels used as catalyst barriers, such as carbon aerogels and graphene aerogels, generally have high electrical conductivity, which compensates for the poor electrical conductivity of semiconductor electrocatalysts. Furthermore, the well-developed pore channels provide a wider exposure area for the catalyst, resulting in more catalytic active sites being involved in the OER [16]. Fu et al. [17] uniformly dispersed Ni/MnO particles in the nanopore channels of 3D graphene aerogel. The large specific surface area and high conductivity resulted in a low OER overpotential of 0.37 V at a current density of 10 mA/cm^2^. Cui et al. [18] loaded ZIF-67 derived hollow NiCo phosphate nanocages onto wastepaper-based carbon aerogel (NCP@WPCA). The NCP@WPCA catalysts exhibited excellent OER activity (0.35 V at 10 mA/cm^2^) due to the enhanced electrochemical surface area. Aerogel-structured catalysts, unlike aerogel catalyst barriers, require a balance between conductivity and catalytic activity. As a result, metallic aerogels and hetero-element-doped carbon aerogels have become a hot research topic [19,20]. The network structure consisting of catalytically active units maximizes the OER area. In addition, the synergistic interactions between transition metals and nonmetal elements significantly adjust the electronic structures, and hence, the binding energy of the intermediates and the active sites for the OER process [21]. Lu et al. [22] embedded nanoscale nickel-cobalt-iron alloys onto conductive boron and nitrogen co-doped/biomass-derived carbon aerogels. The synthesized electrocatalysts exhibited the lowest overpotential (0.32 V at 10 mA/cm^2^) under calcination at 600 °C. Zhang et al. [23] directed the casting of carbon aerogels with honeycomb structure, N and S double-doped and loaded with FeCo alloy nanoparticles (NSCA/FeCo). The interaction between the bimetallic alloy and the double-doped carbon resulted in excellent OER activity of NSCA/FeCo (0.34 V at 10 mA/cm^2^). However, as is well acknowledged, few reports on graphene aerogel electrocatalysts co-doped with metallic and non-metallic elements have been published. On the other hand, the most mature aerogel preparation process is the sol-gel method assisted by supercritical drying, but the expensive price and the complicated process also limit the application of aerogel. To solve the above problems, many new techniques have been proposed in recent years, such as chemical reduction self-assembly, the template method and 3D printing. Meanwhile, atmospheric pressure drying and freeze-drying technologies are also developing rapidly and gradually replacing supercritical drying [13,14,15]. Among the graphene aerogel preparation methods, freeze-drying assisted by chemical reduction self-assembly is undoubtedly the simplest and most efficient method to achieve large-scale production by using the property of the self-assembly of graphene sheet layers into three-dimensional structures. However, further improvements are still needed to achieve the harmonization of performance and structure.

In this work, we anchor metallic cobalt in nitrogen-doped graphene aerogels by chemical reduction and vacuum freeze-drying. It is found that 0.5 wt% of cobalt-anchored and 1 wt% of nitrogen-doped graphene (Co-N-C) aerogel electrocatalyst has an optimal OER overpotential of 0.383 V at a current density of 10 mA/cm^2^. It is noteworthy that the OER overpotential of the Co-N-C aerogel electrocatalyst is significantly lower than that of the commercial RuO_2_ at a current density of 20 mA/cm^2^. In addition, we have also prepared a series of M-N-C aerogel electrocatalysts (M = Mn, Fe, Ni, Pt, Au, etc.) by a similar route, among which Co-N-C has the optimal OER activity. Meanwhile, electronic structures are revealed by DFT calculations for Fe-N-C, Co-N-C, and Ni-N-C aerogel electrocatalysts to explain the catalytic activity and mechanism pathways, keeping consistent with the OER activity results.

## 2. Results and Discussion

### 2.1. Chemical Composition and Structural Analysis

**Figure 1:**
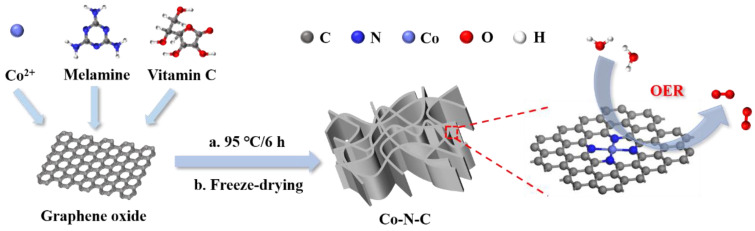
Schematic illustration of the preparation process of the Co-N-C aerogel electrocatalyst.

The preparation process of the Co-N-C aerogel electrocatalyst is shown in Figure 1. In brief, graphene oxide (GO), cobalt chloride (CoCl_2_), melamine, and vitamin C (VC) are mixed in the desired proportions and the Co-N-C aerogel electrocatalyst is obtained by chemical reduction at a reaction temperature of 95 °C and via further vacuum freeze-drying. In our synthetic strategy, the graphene aerogel inherits the abundant vacancies of GO, which can be used for the embedding of nitrogen and metal atoms [24,25]. Meanwhile, both GO and metal ions can be reduced by VC as a strong reductant [26]. It is found that the Co (0.5 wt%)-N (1 wt%)-C aerogel electrocatalyst has the optimal OER activity at a current density of 10 mA/cm^2^, as shown in Figure S1.

**Figure 2:**
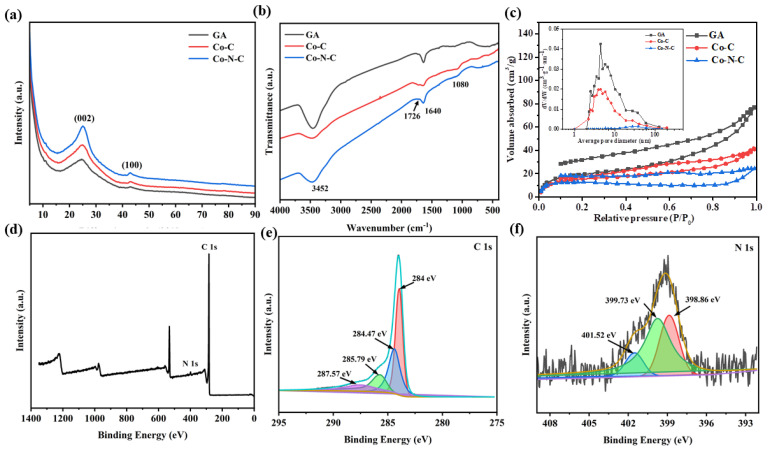
(**a**) XRD, (**b**) FT-IR, and (**c**) N_2_ adsorption–desorption isotherms and the BJH pore size distributions of GA, Co-C, and Co-N-C. XPS (**d**) survey, (**e**) C 1s, and (**f**) N 1s spectrum of GA, Co-C, and Co-N-C.

Overall, structural characterization and OER activity of the preferably selected Co-N-C (0.5 wt% Co and 1 wt% N), Co-C (0.5 wt% Co), and GA are performed. Figure 2a shows the X-ray diffractometer (XRD) patterns of GA, Co-C, and Co-N-C. The two diffraction peaks at 2θ = 25° and 43° can be attributed to the (002) and (100) planes of reduced graphene oxide, respectively [27]. As shown in the Fourier transform infrared absorption (FT-IR) spectra of Figure 2b, the band at 3452 cm^−1^ corresponds to the stretching vibration of O-H, which may be attributed to the adsorption of water under the air atmosphere. The band at 1640 cm^−1^ corresponds to the stretching vibration of the aromatic C=C. The two weak bands at 1726 cm^−1^ and 1080 cm^−1^ belong to the stretching vibrations of C=O and C-O, respectively, which indicates that graphene oxide has been mostly reduced [28]. Unfortunately, probably due to the low content of Co and N, both of them are not detected by the XRD pattern and FT-IR spectra. However, the presence of N is detected in further X-ray photoelectron spectroscopy (XPS) (Figure 2d and Figure S2a). After the charge calibration of the XPS spectrum, the high-resolution spectrum of C1s can be fitted with four peaks. In Figure 2e, the four peaks at 284 eV, 284.47 eV, 285.79 eV, and 287.57 eV correspond to the sp^2^ hybridized carbon of graphene, C-O bond, C-N bond, and C=O bond, respectively [29]. Meanwhile, the high-resolution XPS spectrum of N 1s is composed of three fitted peaks at 398.86 eV, 399.73 eV, and 401.52 eV (Figure 2f), which can be assigned to the pyridine nitrogen, pyrrole nitrogen, and graphite nitrogen, respectively [30]. In addition, the high-resolution spectra of C1s and O1s of GA, Co-C, and Co-N-C aerogel electrocatalysts are compared, and the results of the comparison are shown in Figure S3. The high-resolution spectra of O1s of Co-N-C electrocatalysts can be fitted as two peaks at 530.83 eV (O-C) and 532.47 eV (O=C). Meanwhile the fitted peaks of C1s and O1s of all three electrocatalysts have a little displacement, which may be due to the introduction of Co and N changing the electronic environment of C and O in the electrocatalyst [11]. According to Figure 2c, the type IV isotherm indicates that the pore structure of the sample is heterogeneous with the coexistence of mesopores and macropores. In addition, the hysteresis loop of type H4 reveals that there are narrow fractured pores in the sample. The Barrett–Joiner–Halenda (BJH) pore size distribution diagram also shows that the pore structure of the electrocatalyst is multilevel. It is noteworthy that all isotherms are not closed, which is probably since graphene aerogels are flexible and undergo a slight expansion during the desorption process [31]. The detailed pore structure data are shown in Table 1. The introduction of Co and N elements may hinder the cross-linked assembly of graphene aerogels. The macropores replace some of the mesopores, resulting in a slight decrease in specific surface area, pore size, and pore volume. However, the coherent pore structure also exposes more active sites, which is beneficial to enhancing the OER activity.

**Figure 3:**
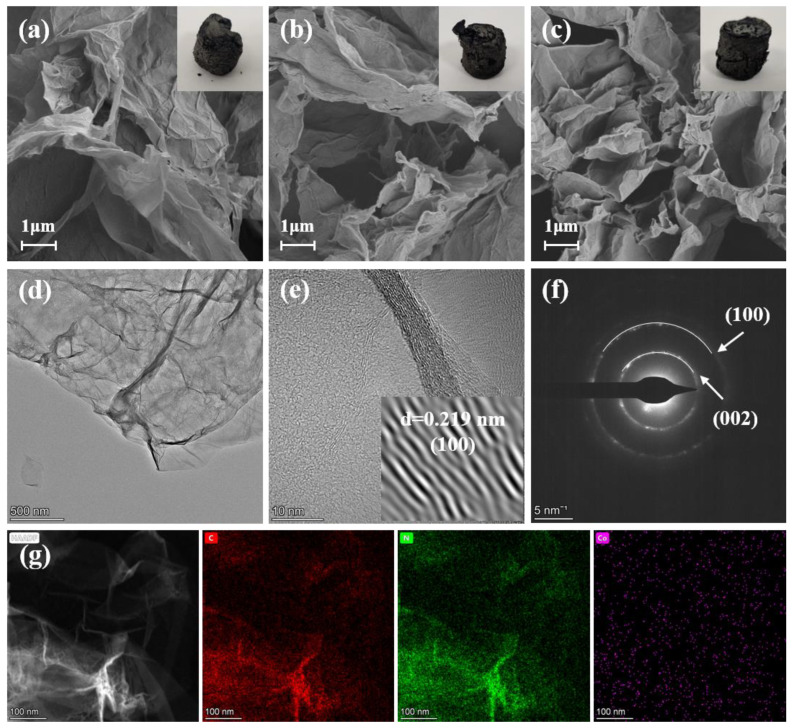
(**a**–**c**) SEM and physical photographs of GA, Co-C, and Co-N-C, respectively. (**d**,**e**) TEM, (**f**) SAED, and (**g**) mappings of Co-N-C.

Figure 3 shows the microstructure and elemental distribution of GA, Co-C, and Co-N-C. Figure 3a–c shows the scanning electron microscope (SEM) images of GA, Co-C, and Co-N-C, respectively, and the insets are the corresponding digital photographs. It can be seen that the introduction of Co and N elements does not have a big impact on the structure of the aerogel, which is visualized as a three-dimensional porous structure assembled by graphene sheet layers. The structure is predominantly macroporous, which is consistent with the results of nitrogen adsorption and desorption tests. The lamellar structure of graphene can be seen in Figure 3d, and the lattice stripes attributed to the (100) plane of graphene can be distinguished at a higher magnification (Figure 3e). In Figure 3f, the selected area electron diffraction (SAED) pattern of Co-N-C has two clear concentric rings that can correspond to the (100) and (002) planes of graphene, respectively, which is consistent with the XRD results. As shown in Figure 3g, the distribution ranges of element C and element N are highly overlapping in the mapping diagram of Co-N-C. It is noteworthy that the Co element is uniformly distributed in the sample, with an amount of 0.41 wt% detected in the energy dispersive spectroscopy (EDS) of Figure S2b, which is strong proof of the presence of the Co element in the sample.

### 2.2. OER Performance Analysis

**Figure 4:**
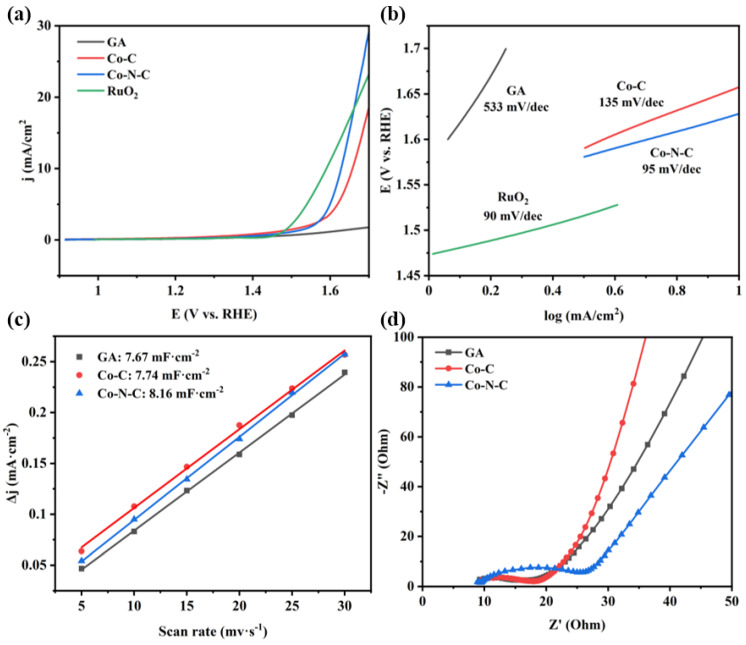
(**a**) LSV, (**b**) the Tafel curves, (**c**) current density differences plotted against the scan rate, and (**d**) EIS for GA, Co-C, and Co-N-C.

Overpotential at a certain current density is an effective technique to evaluate the OER activity of electrocatalysts. As shown in Figure 4a, the Co-N-C has the lowest reaction potential of 1.62 V at a current density of 10 mA/cm^2^. Based on the water oxidation potential of 1.23 V and the IR compensation potential of 0.007 V (I_0_ = 0.7 mA, R_ct_ = 10 Ω), the overpotential of Co-N-C aerogel electrocatalyst can be obtained as about 0.383 V, which is lower than that of many reported Co-N-C electrocatalysts (Table S1). Notably, the overpotential of Co-N-C is lower than that of the noble metal electrocatalyst (RuO_2_) when the current density is increased to 20 mA/cm^2^. In addition, a series of M-N-C aerogel electrocatalysts (M = Mn, Fe, Ni, Pt, Au, etc.) are prepared by a similar route, among which Co-N-C has the optimal OER activity, as shown in Figure S4. The Tafel slope of Co-N-C (95 mV/dec) is also significantly lower than that of Co-C (135 mV/dec) and GA (533 mV/dec), and the lower slope is more favorable for the catalytic reaction (Figure 4b). Figure 4c and Figure S5 demonstrate the electrochemical surface area of aerogel electrocatalysts. Based on the working electrode surface area of 0.07 cm^2^, the double-layer capacitances of GA, Co-C, and Co-N-C aerogel electrocatalysts are 0.5369, 0.5418, and 0.5712 mF, respectively. The corresponding electrochemical surface areas are 8.95, 9.03, and 9.52 cm^2^. Co-N-C has higher electrochemical surface areas than those of GA and Co-C. In addition, a stability test was performed on a Co-N-C aerogel electrocatalyst, as shown in Figure S6. By comparing the chronopotentiometric curve at a current density of 10 mA/cm^2^, it can be found that the electrocatalyst has excellent stability with a stable reaction potential of 1.62 V within 5 h. Although the Co-N-C aerogel electrocatalyst has the largest impedance in the EIS spectrum of Figure 4d, it is significantly smaller than that of other structures of Co-N-C electrocatalysts that have been reported so far [32].

### 2.3. Theoretical Calculations

**Figure 5:**
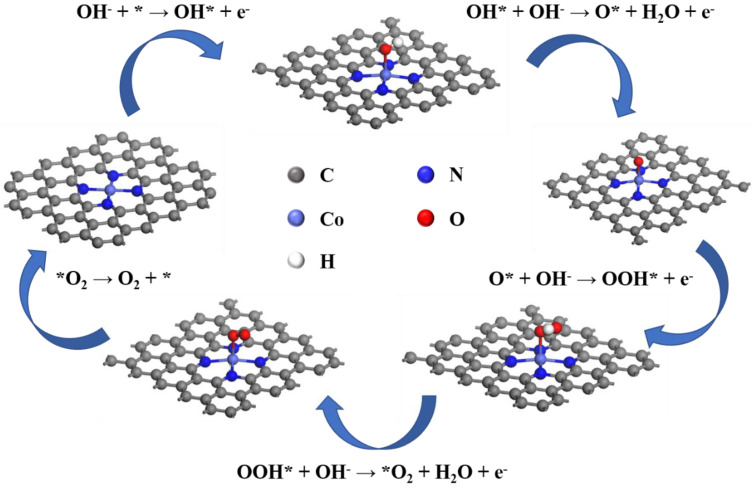
Structural diagrams of Co-N-C for each primitive process of OER (alkaline conditions).

Based on the electrochemical experimental results, Co-N-C, Fe-N-C, and Ni-N-C aerogel electrocatalysts are selected for DFT calculations. Taking the Co-N-C aerogel electrocatalyst as an example, Figure 5 shows a schematic diagram of the structural changes of the electrocatalyst during the OER transition through six stages. It should be noted that the *O_2_ intermediate formation introduced in this calculation is a thermocatalytic primitive step and does not involve the transfer of electrons and proton pairs [33]. From the calculations, it is known that the total Gibbs free energy change of the reaction is 4.92 eV both for Co-N-C, Fe-N-C, and Ni-N-C aerogel electrocatalysts, which is thermodynamically determined.

**Figure 6:**
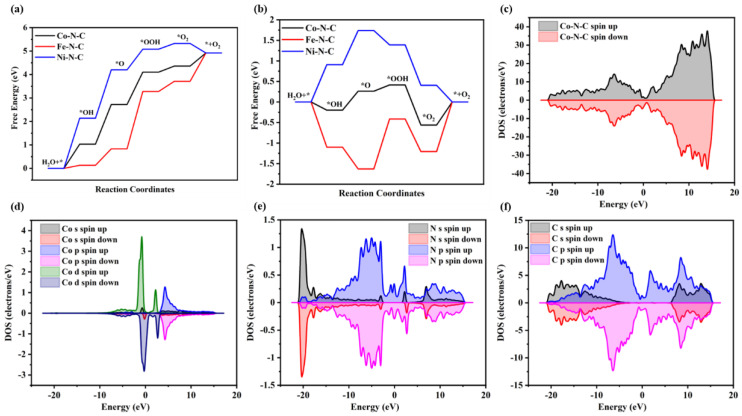
(**a**) Gibbs free energy variation in different aerogels for each of the OER primitive processes. (**b**) Gibbs free energy variation in different aerogels in each primitive process of OER (U = 1.23 V). (**c**) The total density of states, (**d**) partial density of the state of Co atom, (**e**) partial density of the state of N atom, and (**f**) partial density of the state of C atom of the Co-N-C.

As seen in Figure 6a, the Gibbs free energies of the five-step reaction of Ni-N-C are 2.14 eV, 2.06 eV, 0.88 eV, 0.24 eV, and −0.40 eV, which means that the rate-determining step is the first reaction with the largest change in Gibbs free energy. It indicates that the adsorbed state *OH formed by the release of one electron from the H_2_O molecule is not easily bound to the polished surface of the catalyst. Similarly, the Gibbs free energies for the five-step reaction of Co-N-C are 1.02 eV, 1.69 eV, 1.36 eV, 0.25 eV, and 0.58 eV, which implies that the rate-determining step is the second reaction step. It indicates that *OH can exist stably on the surface of Co-N-C, but it is difficult to form the adsorbed state *O by releasing hydrogen ions and electrons. For Fe-N-C, the Gibbs free energies of the five-step reaction are 0.60 eV, 0.74 eV, 2.25 eV, 0.39 eV and 0.90 eV, respectively. It means that the rate-determining step is the third reaction step, implying that it is difficult for the *O formed on the catalyst surface to release protons and electrons to form the adsorbed *OOH by reacting with H_2_O molecules. Figure 6b shows the Gibbs free energy change of several different aerogel electrocatalysts at U = 1.23 V for different primitive processes. At this point, if the Gibbs free energy change of each reaction step is positive, the value is equal to the overpotential of the reaction at that step. If the Gibbs free energy of the step is negative, each step of the primitive reaction at that voltage is spontaneous and no additional work is required to be input. From Figure 6b, it can be seen that the overpotential is 0.462 eV, 0.909 eV, and 1.215 eV for Co-N-C, Fe-N-C, and Ni-N-C, respectively. Therefore, it can be inferred that the Co-N-C aerogel electrocatalyst has the optimal OER activity, which is consistent with the electrochemical performance results.

**Figure 7:**
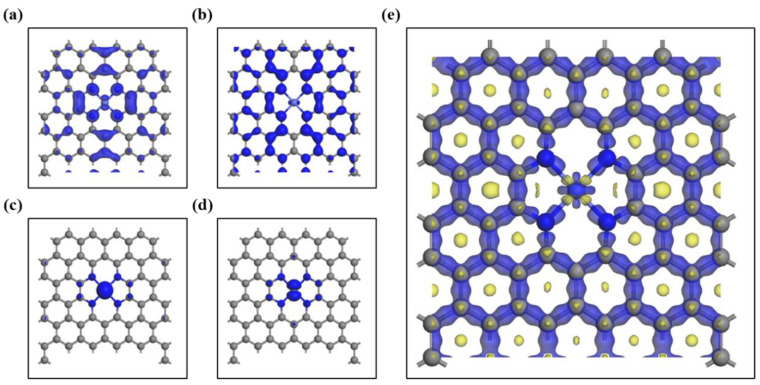
Schematic illustration of (**a**) the top of the valence band and (**b**) the bottom of the conduction band for spin-up electrons. Schematic illustration of (**c**) the top of the valence band and (**d**) the bottom of the conduction band for spin-down electrons. (**e**) Illustration of the electron density difference iso-surface for Co-N-C (blue region represents the accumulation of electrons and the yellow region represents the depletion of electrons).

To further investigate the electronic structure changes, the total density of states (TDOS in Figure 6c) of Co-N-C aerogel electrocatalysts and the partial density of state (PDOS) of Co, N, and C atoms have been calculated. As shown in Figure 6d, the spin-up and spin-down electrons are asymmetric along with the energy levels, with the d-orbital electrons induced by the Co atom occupying most of the top of the valence band and the spin-down d-orbital electrons occupying most of the bottom of the conduction band. Furthermore, according to Figure 6e,f, both C and N atoms in the N-doped graphene lattice show symmetric spin-up and spin-down electrons, and the p-orbital electrons have a contribution to the Fermi energy level of the electrocatalyst. It is noteworthy that the s-orbital electrons of the N atom are slightly asymmetric near the Fermi energy level, which may be the influence of the N atom by the Co atom close to it. In the PDOS of the electrocatalyst (Figure 6d–f), the PDOS of Co, N, and C atoms near the Fermi energy level overlap, suggesting that there is an interaction to enhance the catalytic performance of the OER. To better understand the DOS of the electrocatalyst, a schematic illustration of the top of the valence band and the bottom of the conduction band of spin-up and spin-down electrons are constructed, as shown in Figure 7a–d. The spin-up electrons of C and N atoms and the spin-down electrons of Co atoms form the top of the valence band and the bottom of the conduction band of the electrocatalyst. The electron density difference of the electrocatalyst is further investigated for the interactions between Co, N, and C elements. It can be seen from Figure 7e that the electrons are mainly concentrated in the chemical bond and the electrons on the Co atom migrate towards the N atom. In addition, the electron distribution on the Co atom is consistent with the stretching direction of the 3d orbital of the Co atom [34].

## 3. Conclusions

In summary, cobalt-anchored nitrogen-doped graphene aerogels are prepared by a simple chemical reduction and vacuum freeze-drying. The coherent pore structure and uniformly distributed active sites of the aerogels are favorable for the improvement in electrocatalytic performance. After ratio adjustment and performance testing, Co (0.5 wt%)-N (1 wt%)-C has an optimal OER reaction overpotential (0.383 V at 10 mA/cm^2^), a small Tafel slope of (95 mV/dec), a large ECSA (9.52 cm^2^), and excellent stability. It is noteworthy that the overpotential of Co-N-C is already lower than that of the commercial RuO_2_ at a current density of 20 mA/cm^2^. Moreover, the OER activity of the Co-N-C aerogel electrocatalyst is superior to a series of M-N-C aerogel electrocatalysts (M = Mn, Fe, Ni, Pt, Au, etc.) prepared by a similar route and other Co-N-C electrocatalysts that have been reported. Theoretical calculations are performed for Fe-N-C, Co-N-C, and Ni-N-C aerogel electrocatalysts to derive the Gibbs free energy and the rate-determining step required for each original step of the OER processes. The results confirm that the metal activity trend is Co-N-C > Fe-N-C > Ni-N-C, which is consistent with the OER performance test results. Due to the simple preparation method, abundant raw materials, and superior electrocatalytic performance, Co-N-C aerogel electrocatalysts can be considered to be one of the most promising electrocatalysts for energy storage and energy saving.

## 4. Materials and Methods

### 4.1. Materials

The cobalt chloride (CoCl_2_, 99.5%), vitamin C (VC, 99.0%), deionized water (H_2_O, 99.9%), and melamine (C_3_H_6_N_6_, 99.8%) used in the experiments were all from Sinopharm Chemical Reagent Co., Ltd., Shanghai, China. The graphene oxide aqueous solution (GO, 2 mg/mL) was provided by Suzhou TanFeng Graphene Technology Co., Ltd., Nanjing, China. The above chemicals were used directly as raw materials without further purification.

### 4.2. Method

The resulting Co-N-C aerogel samples were obtained by chemical reduction and vacuum freeze-drying. Different masses of CoCl_2_ and melamine were dissolved into 10 mL of graphene oxide aqueous solution, and then 80 mg of VC was added and stirred for 30 min. The mixed solution was further reacted in an oven at 95 °C for 6 h for the self-assembly of the graphene oxide aqueous solution. The formed wet gel was subsequently washed with deionized water to remove impurities inside the pore structures. Finally, Co-N-C aerogel electrocatalysts were prepared by the vacuum freeze-drying technique. Pristine graphene aerogel (GA) and cobalt-anchored graphene aerogel (Co-C) were also prepared by a similar route.

### 4.3. Characterizations

An X-ray diffractometer (XRD), D8 Advance (Bruker, Bremen, Germany), was used for the physical phase analysis of the composite aerogel materials. A Fourier transform infrared absorption spectrometer (FT-IR), Nicolet iS5 (Thermo Fisher Scientific, Waltham, MA, USA), was used to determine the infrared spectra of the aerogel materials. An X-ray photoelectron spectrometer (XPS), ESCALAB 250XI (Thermo Fisher Scientific, Waltham, MA, USA), was used to analyze the composition of the aerogel materials. A scanning electron microscope model (SEM), Sigma 500 (Zeiss, Oberkochen, Germany), was used to observe the morphology and structure of the aerogel materials. A transmission electron microscope model (TEM), FEI Talos F200S (Thermo Fisher Scientific, Waltham, MA, USA), was used to analyze the microscopic morphology and energy dispersive spectroscopy (EDS) of the aerogel materials. A specific surface area analyzer, V-Sorb 2800P (Gold APP Instruments Co., Beijing, China), was used to determine the specific surface area and pore structure distribution of the aerogel materials.

### 4.4. Electrochemical Measurements

A three-electrode system was used for the experiments, in which Hg/HgO (1 M NaOH) was used as the reference electrode, platinum wire as the counter electrode, glassy carbon electrode loaded with catalyst as the working electrode, and 1 M KOH as the electrolyte. A total of 4 mg of catalyst was homogeneously dispersed in a mixture of 1 mL of ethanol and 40 µL of nafion. A total of 5 μL of catalyst solution was applied dropwise to a 3 mm-diameter glassy carbon electrode (the surface area was about 0.07 cm^2^), which was dried with an infrared baking lamp and used as the working electrode. The electrocatalyst was first activated by cyclic scanning voltammetry (CV) with a scanning potential range of 0–1 V (E vs. Hg/HgO) at a rate of 100 mV/s for 20 cycles. Linear scanning voltammetry (LSV) was performed over a potential range of 0–1 V (E vs. Hg/HgO) at a rate of 5 mV/s. The chronopotentiometric curve was used to indicate the stability of the electrocatalyst with a constant operating current of 0.7 mA (current density of 10 mA/cm^2^). Electrochemical impedance spectroscopy (EIS) was performed with a scan frequency range of 0.01–100,000 Hz and an amplitude of 5 mV without applied voltage. The electrochemical surface area (*ECSA*) was measured with CV over a potential range of 1.1–1.2 V vs. RHE. The double-layer capacitance (*C_dl_*) was estimated by plotting the current density difference (ΔJ = J_a_ − J_c_ at 1.15 V vs. RHE) versus the scan rate. ECSA has a linear relationship with *C_dl_*, as shown in Equation (1) [35].

(1) $$ECSA=\frac{C_{dl}}{60\;\mu F\cdot cm^{-2}}$$

### 4.5. Theoretical Calculations

The electronic structures and Gibbs free energies of aerogel electrocatalysts were calculated by the DFT calculations of the Dmol3 package. For exchange-correlation, the Perdew–Burke–Ernzerhof method with a generalized gradient approximation (PBE-GGA) was utilized. The vacuum layer was set to 15 Å with periodic boundary conditions, which were used throughout the calculation process to ensure that the regularly repeated structures did not interact. The Brillouin zone was sampled using a 4 × 4 × 1 Monkhorst–Pack k-points mesh during the geometry optimization process, and the thresholds were set at 1.0 × 10^−5^ eV/atom, 0.03 eV/Å, 0.05 GPa, and 0.001 Å, respectively. The Grimme method was used to consider the weak van der Waals interaction [24,36].

A typical OER reaction under alkaline conditions was generally considered to be composed of four elementary steps.

(2) $$OH^{-}+*\to OH*+e^{-}$$

(3) $$OH*+OH^{-}\to O*+H_{2}O+e^{-}$$

(4) $$O*+OH^{-}\to OOH*+e^{-}$$

(5) $$OOH*+OH^{-}\to O_{2}+H_{2}O+e^{-}+*$$

The reaction involved three intermediates in the adsorbed state, *OH, *O, and *OOH. In addition, each of the four steps involves the coupling transfer of one electron and one proton. Therefore, the calculation of the Gibbs free energy for each elementary step could be performed using Norskov’s standard hydrogen electrode approximation [37]. In addition, according to Equation (6), for an ideal OER electrocatalyst at pH = 14, the Gibbs free energy for each step of the primitive reaction was 0.401 eV. In fact, the conclusion of the rate control step does not change regardless of the acidic or basic conditions, but only the same amount was added or subtracted to each step of the elementary reaction at the same time.

(6) $$G(H^{+})+G(e^{-})=0.5G_{H_{2}}-0.0592pH$$

## Figures and Tables

**Table 1:** Pore structures of GA, Co-C, and Co-N-C.

Catalyst	Micropore Pore Volumes (cm^3^/g)	BET Specific Surface Areas (m^2^/g)	BJH Adsorption Average Pore Diameters (nm)	BJH Adsorption Pore Volumes (cm^3^/g)
GA	0.12	69.73	6.81	0.11
Co-C	0.06	56.29	4.51	0.05
Co-N-C	0.04	42.93	3.50	0.03

## Data Availability

The raw/processed data required to reproduce these findings cannot be shared at this time as the data also form part of an ongoing study.

## Supplementary Materials

The following supporting information can be downloaded at: https://www.mdpi.com/article/10.3390/gels9040313/s1, Figure S1. (a) LSV curves of Co-C with different cobalt doping amounts (wt%); (b) LSV curves of Co-N-C with different nitrogen doping amounts (wt%). Figure S2. (a) Co 2p high-resolution XPS spectrum and (b) EDS of Co-N-C. Figure S3. C1s (a) and O1s (b) high-resolution XPS spectrum of GA, Co-C, and Co-N-C. Figure S4. LSV curves of M-N-C aerogel electrocatalysts (M = Mn, Fe, Ni, Pt, Au, etc.). Figure S5. Typical CV curves of (a) GA, (b) Co-C, and (c) Co-N-C at different scan rates. Figure S6. The chronopotentiometric curve for the Co-N-C at 10 mA/cm^2^. Table S1. Comparison of Co-N-C aerogel and some previously reported Co-N-C electrocatalysts in terms of OER performance. References [38,39,40,41,42,43,44,45,46,47,48,49,50,51] are cited in the supplementary materials.
