# Supplementary material for: Tuning the Electronic Structure of a Novel 3D Architectured Co-N-C Aerogel to Enhance Oxygen Evolution Reaction Activity
Source: Gels. 2023 Apr 7;9(4):313. doi: 10.3390/gels9040313 (PMC10137415; doi:10.3390/gels9040313)
Supplement: Supplementary file 1 [file gels-09-00313-s001.zip › gels-2310331-supplementary.pdf]

Supplementary Information

Tuning the Electronic Structure of a Novel 3D Architectured Co-N-C Aerogel to Enhance Oxygen Evolution Reaction Activity

Chunsheng Ni [1], Shuntian Huang [1], Tete Daniel Koudama [1], Xiaodong Wu [1,*], Sheng Cui [1,*], Xiaodong Shen [1] and Xiangbao Chen [2]

[1] College of Materials Science and Engineering, Nanjing Tech University, Nanjing 210009, China
[2] AECC Beijing Institute of Aeronautical Materials, Beijing 100095, China
* Correspondence: wuxiaodong@njtech.edu.cn (X.W.); scui@njtech.edu.cn (S.C.)

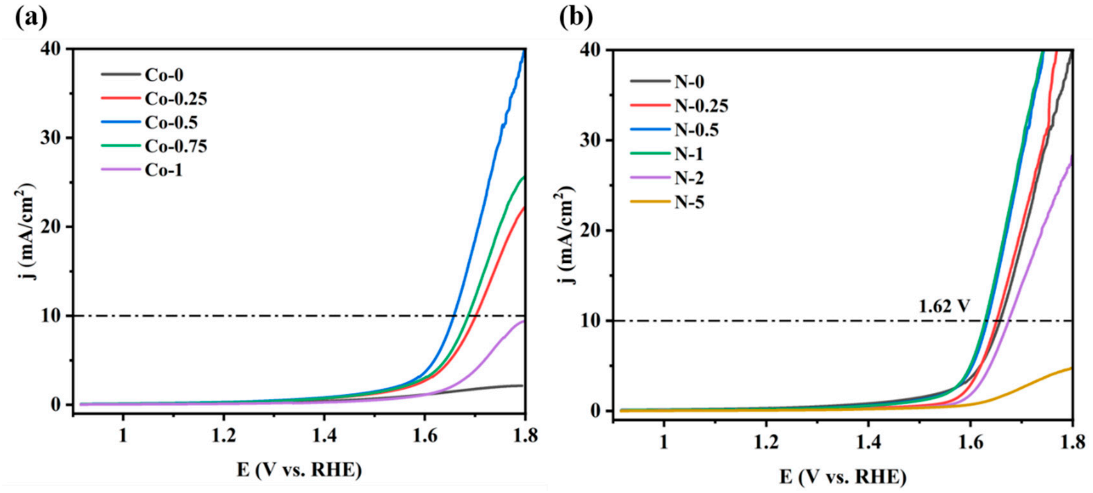

Figure S1. (a) LSV curves of Co-C with different cobalt doping amounts (wt%); (b) LSV curves of Co-N-C with different nitrogen doping amounts (wt%).

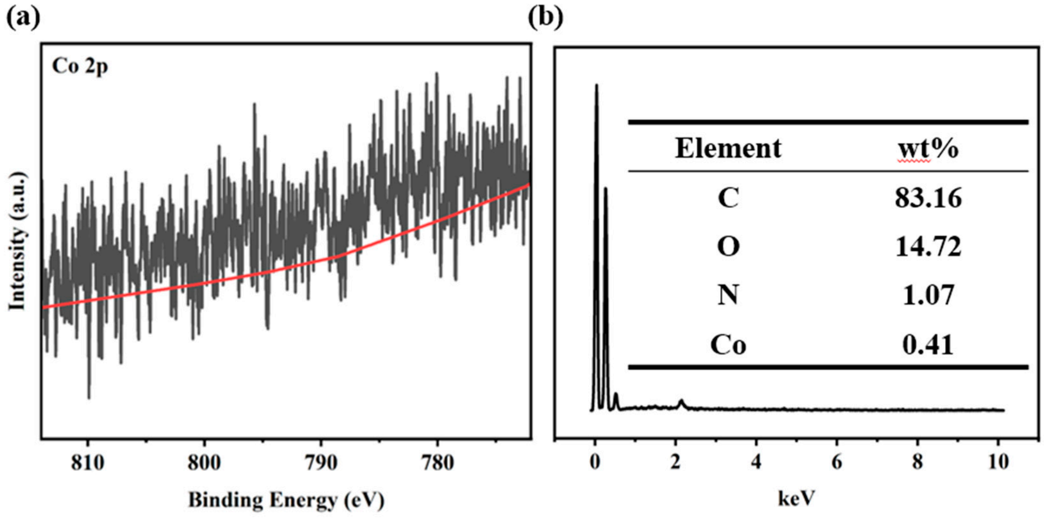

Figure S2. (a) Co 2p high-resolution XPS spectrum and (b) EDS of Co-N-C.

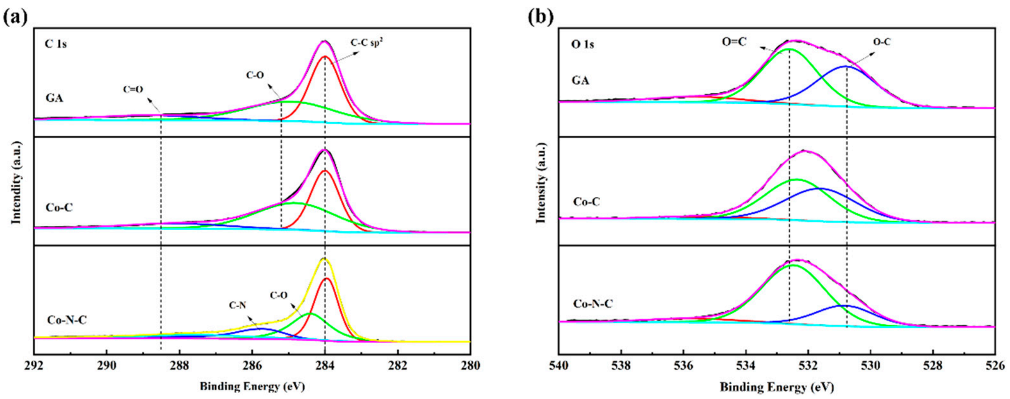

Figure S3. C 1s (a) and O 1s (b) high-resolution XPS spectrum of GA, Co-C, and Co-N-C.

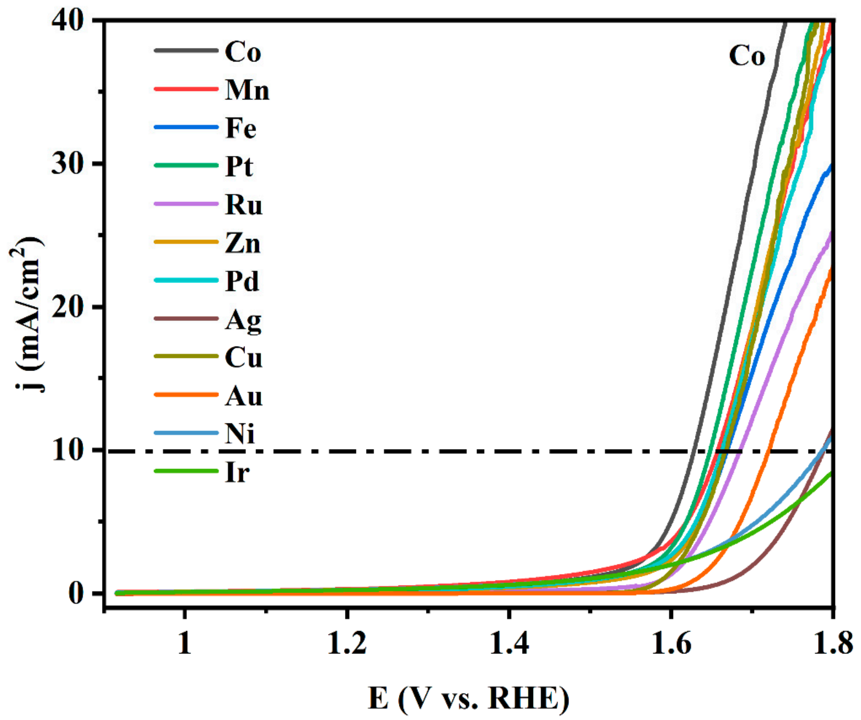

Figure S4. LSV curves of M-N-C aerogel electrocatalysts (M=Mn, Fe, Ni, Pt, Au, etc.).

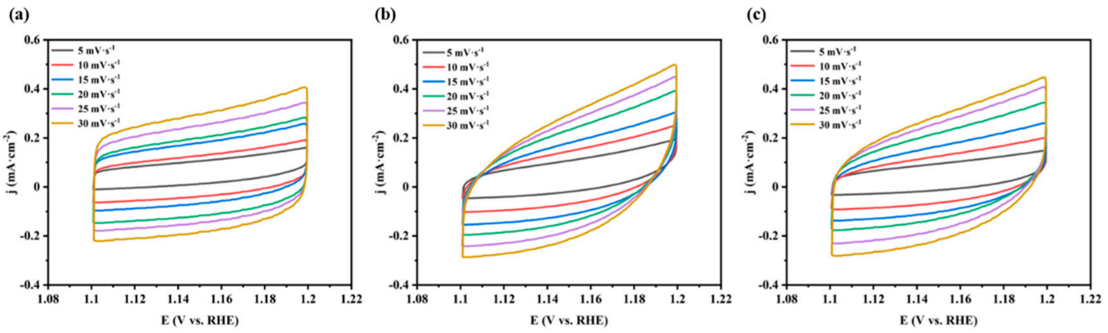

Figure S5. Typical CV curves of (a) GA, (b) Co-C, and (c) Co-N-C at different scan rates.

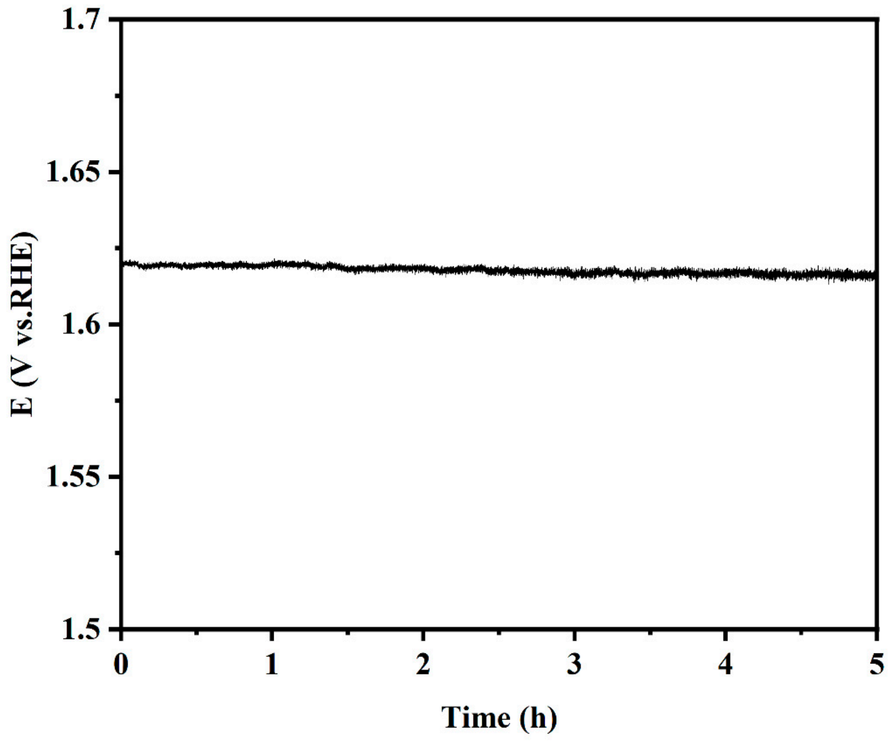

Figure S6. Chronopotentiometric curve for the Co-N-C at 10 mA/cm^2.

Table S1. Comparison of Co-N-C aerogel and some previously reported Co-N-C electrocatalysts in terms of OER performance.

electrocatalyst	Overpotential at 10mA/cm^2	Reference
Co-N-C aerogel	0.383 V	This work
Mo_2C-Co-N-C	0.396 V	[1]
Co@CoO@Co_3O_4-N/C	0.45 V	[2]
Co/Co-N-C	0.41 V	[3]
3D Co-N-C NN-800	0.47 V	[4]

CoO_x/Co-N-C	0.42 V	[5]
3D-Co–N–C-annealing	0.42 V	[6]
3DOM Co-NCPs-900	0.418 V	[7]
Mn/Co–N–C-0.02-800	0.43 V	[8]
CoO/Co@N–C	0.442 V	[9]
Co–N–C-0.4	0.39 V	[10]
Fe,Co,N–C	0.41 V	[11]
Co/N-Pg	0.40 V	[12]
CoA@CNC-700	0.46 V	[13]
Co-NCNT	0.40 V	[14]

References

1. Sam, D.K.; Gong, S.H.; Durairaj, A.; Sam, E.K.; Liu, J.; Lv, X.M. Fabrication of highly dispersed Mo2C coupled with Co-N-C via self-template as bifunctional electrocatalysts. *Int. J. Energy Res.* **2021**, 45, 10989-11001.
2. Xu, G.; Xu, G.C.; Ban, J.J.; Zhang, L.; Lin, H.; Qi, C.L.; Sun, Z.P.; Jia, D.Z. Cobalt and cobalt oxides N-codoped porous carbon derived from metal-organic framework as bifunctional catalyst for oxygen reduction and oxygen evolution reactions. *J. Colloid Interf Sci.* **2018**, 521, 141-149.
3. Wang, D.; Yang, P.X.; Xu, H.; Ma, J.Y.; Du, L.; Zhang, G.X.; Li, R.P.; Jiang, Z.; Li, Y.; Zhang, J.Q.; An, M.Z. The dual-nitrogen-source strategy to modulate a bifunctional hybrid Co/Co-N-C catalyst in the reversible air cathode for Zn-air batteries. *J. Power Sources* **2021**, 485, 229339.
4. Wang, R.; Cao, J.Y.; Cai, S.C.; Yan, X.M.; Li, J.S.; Yourey, W.M.; Tong, W.; Tang, H.L. MOF@Cellulose Derived Co-N-C Nanowire Network as an Advanced Reversible Oxygen Electrocatalyst for Rechargeable Zinc-Air Batteries. *Acs Appl. Energ. Mater.* **2018**, 1, 1060-1068.
5. Xin, W.L.; Lu, K.K.; Zhu, D.R.; Zeng, H.B.; Zhang, X.J.; Marks, R.S.; Shan, D. Highly reactive N,N '-carbonyldiimidazole-tailored bifunctional electrocatalyst for oxygen reduction and oxygen evolution. *Electrochim. Acta* **2019**, 307, 375-384.
6. Xiang, F.; Yang, J.; Gong, W.X.; Zou, J.; Liu, Y.Z.; Li, Y.L.; Guo, H.; Wang, L.P.; Niu, X.B. Facile Synthesis of Graphene-like Porous Carbon with Densely Populated Co-N-x Sites as Efficient Bifunctional Electrocatalysts for Rechargeable Zinc-Air Batteries. *Acs Appl. Energ. Mater.* **2021**, 4, 11545-11554.
7. Hao, Y.X.; Kang, Y.M.; Mi, Y.J.; Wang, W.; Lei, Z.Q. Highly ordered micro-meso-macroporous Co-N-doped carbon polyhedrons from bimetal-organic frameworks for rechargeable Zn-air batteries. *J. Colloid Interf Sci.* **2021**, 598, 83-92.
8. Wei, L.C.; Qiu, L.J.; Liu, Y.Y.; Zhang, J.M.; Yuan, D.S.; Wang, L. Mn-Doped Co-N-C Dodecahedron as a Bifunctional Electrocatalyst for Highly Efficient Zn-Air Batteries. *ACS Sustain. Chem. Eng.* **2019**, 7, 14180-14188.
9. Ding, Y.J.; Yang, W.Y.; Gao, S.; Sun, W.Z.; Sun, C.X.; Li, Q. Strongly Cooperative Nano-CoO/Co Active Phase in Hierarchically Porous Nitrogen-Doped Carbon Microspheres for Efficient Bifunctional Oxygen Electrocatalysis. *Acs Appl. Energ. Mater.* **2020**, 3, 1328-1337.
10. Hu, E.L.; Ning, J.Q.; He, B.; Li, Z.P.; Zheng, C.C.; Zhong, Y.J.; Zhang, Z.Y.; Hu, Y. Unusual formation of tetragonal microstructures from nitrogen-doped carbon nanocapsules with cobalt nanocores as a bi-functional oxygen electrocatalyst. *J. Mater. Chem. A.* **2017**, 5, 2271-2279.
11. Sarkar, S.; Biswas, A.; Siddharthan, E.E.; Thapa, R.; Dey, R.S. Strategic Modulation of Target-Specific Isolated Fe,Co Single-Atom Active Sites for Oxygen Electrocatalysis Impacting High Power Zn-Air Battery. *Acs Nano* **2022**, 16, 7890–7903.

12. Tian, Y.H.; Xu, L.; Bao, J.; Qian, J.C.; Su, H.N.; Li, H.M.; Gu, H.D.; Yan, C.; Li, H.N. Hollow cobalt oxide nanoparticles embedded in nitrogen-doped carbon nanosheets as an efficient bifunctional catalyst for Zn-air battery. *J. Energy Chem.* **2019**, 33, 59-66.
13. Shen, M.X.; Gao, K.; Xiang, F.K.; Wang, B.B.; Dai, L.; Zheng, L.Q.; Baker, F.; Duan, C.; Zhang, Y.H.; Sun, S.H.; Ni, Y.H. Nanocellulose-assisted synthesis of ultrafine Co nanoparticles-loaded bimodal micro-mesoporous N-rich carbon as bifunctional oxygen electrode for Zn-air batteries. *J. Power Sources* **2022**, 450, 227640.
14. Pei, Z.X.; Huang, Y.; Tang, Z.J.; Ma, L.T.; Liu, Z.X.; Xue, Q.; Wang, Z.F.; Li, H.F.; Chen, Y.; Zhi, C.Y. Enabling highly efficient, flexible and rechargeable quasi-solid-state zn-air batteries via catalyst engineering and electrolyte functionalization. *Energy Storage Mater.* **2019**, 20, 234-242.
